# NH_4_F and VO (Acac)_2_ Tuning of Hexagram-Shaped Co_3_O_4_ Morphology for High-Performance Supercapacitor Electrodes

**DOI:** 10.3390/nano16030162

**Published:** 2026-01-26

**Authors:** Huanping Yang, Zhiguo Zhang, Ziming Fang, Yutian Zhao, Bitao Xiong, Xiaoli Lang, Yanting Shen, Xing’ao Li, Yan Wang

**Affiliations:** 1 School of Science, Zhejiang University of Science and Technology, No. 318, Liuhe Road, Xihu District, Hangzhou 310023, China; 2 Tsing Bosch (Zhuhai) Technology Co., Ltd., Zhuhai 519000, China; 3 School of Textile Science and Engineering, Xi’an Polytechnic University, No. 58, Shan Gu Avenue, Lintong District, Xi’an 710600, China; 4 New Energy Technology Engineering Laboratory of Jiangsu Province, School of Science, Nanjing University of Posts and Telecommunications (NJUPT), Nanjing 210023, China

**Keywords:** NH_4_F, VO (acac)_2_, Co_3_O_4_, hexagram structure, cycling performance

## Abstract

In this work, by employing NH_4_F as a structure-directing agent (SDA) and VO(acac)_2_, we have manipulated the morphology of Co_3_O_4_, leading to the creation of a novel hexagram-like structure with exceptional evenness in distribution. To comprehend the growth mechanism and elucidate the functions of various agents involved, experiments were conducted under diverse conditions with varying reagent ratios. The results indicate that, under the influence of NH_4_F as the structure-directing agent (SDA), the hexagram-shaped Co_3_O_4_ structure exhibits sensitivity to both reaction time and temperature, implying that its growth mechanism is regulated by the Kirkendall effect and involves partial cation exchange. Additionally, with alteration of reagent ratios, Co_3_O_4_ with ball-flower morphology was synthesized successfully. Through cross-section SEM examination, the observed growth mechanisms for both the hexagram and ball-flower structures were substantiated. Lastly, electrochemical performance tests of the hexagram and ball-flower structures on SC electrode were carried out, and specific capacitances were 452 C/g (1062 F/g) and 696 C/g (1339 F/g), respectively. The hexagram-shaped Co_3_O_4_ structure displays exceptional SC electrode material characteristics, retaining an outstanding capacitance of 93.1% even after 10,000 cycles, highlighting its superior cycle performance. This paper hopes to inspire further SC electrode materials studies based on its novel morphology modulation strategy.

## 1. Introduction

With China’s recently announced carbon-peaking and carbon-neutrality targets, the development of efficient energy-storage technologies has become increasingly important. Among these technologies, supercapacitors (SCs) have attracted substantial attention due to their high power density, long cycle life, and environmental compatibility. Several studies on SC electrode materials are proposed, and among them transition metal oxides (TMOs) have received extensive attention due to their high theoretical specific capacitances and applicability. The theoretical capacitance of Co_3_O_4_ is approximately 3560 F g^−1^, based on its faradic redox reactions [1]. Also, Co_3_O_4_ is facing scrutiny for SC electrodes due to its changeable morphology, including nanowire, nanorod, and nanosheet forms, whose electrochemical performance is entwined inextricably with their structures and morphologies [2,3].

Structure-directing agents (SDAs) play a crucial role in tailoring the morphology of transition metal oxides (TMOs), thereby influencing their electrochemical properties. Common SDAs such as urea, CTAB (cetyltrimethylammonium bromide), PVP (polyvinylpyrrolidone), and NH_4_F (ammonium fluoride) regulate nucleation and crystal growth through different mechanisms. Urea slowly releases OH^−^ during hydrothermal reactions, enabling controlled precipitation and formation of hierarchical or hollow structures. CTAB, as a cationic surfactant, directs anisotropic growth by forming micelle templates that regulate surface energy and facet exposure. PVP acts as a polymeric capping agent, selectively adsorbing onto crystal planes and preventing particle agglomeration, which results in well-dispersed nanostructures. NH_4_F provides F^−^ ions that complex with metal cations, induce slow nucleation, and promote the formation of hexagonal or plate-like architectures. Together, these SDAs offer powerful strategies for morphology engineering in TMOs, enabling enhanced ion transport, larger active surface area, and improved electrochemical performance. NH_4_F is an SDA that is widely used to regulate morphology of TMOs by tuning the pH value of solutions and releasing fluorine ions [4,5,6,7,8]. Several research proposals focus on optimizing the electrochemical performance of batteries and SCs through strategic modulation of Co_3_O_4_ morphology. Liu et al. successfully synthesized Co_3_O_4_ nanowires and hexagonal nanosheets wherein NH_4_F induced alterations in the product’s morphology via pH manipulation of the solution [9]. Nanorods and gear-like nanosheets of Co_3_O_4_ were synthesized by Zhou using NH_4_F. The study revealed that elevated dosages of NH_4_F led to increased crystallinity and decreased specific surface area of Co_3_O_4_ [10]. Needle Co_3_O_4_ grown on carbon fibers to form a core-shell structure was reported by Liu [11]. The growing mechanisms of novel structures inspire innovative morphology modulation strategies that might be conducive to improving the electrochemical performance of SCs by tuning the morphologies of promising materials.

In this work, by employing NH_4_F as a structure-directing agent (SDA), as well as VO(acac)_2_, we have manipulated the morphology of Co_3_O_4_, leading to the creation of a novel hexagram-like structure with exceptional evenness of distribution. To comprehend the growth mechanism and elucidate the functions of the various agents involved, experiments were conducted under diverse conditions and with varying reagent ratios. Additionally, with the alteration of reagent ratios, Co_3_O_4_ with ball-flower morphology was also synthesized successfully. Lastly, electrochemical performance tests of the hexagram and ball-flower structures on SC electrode were carried out, and the hexagram-shaped Co_3_O_4_ structure displays exceptional SC electrode material characteristics, highlighting its superior cycle performance. This paper hopes to inspire further SC electrode materials studies based on its novel morphology modulation strategy.

## 2. Experimental Section

### 2.1. Preparation of the Co_3_O_4_ Hexagram Structure

NH_4_F supplies F^−^, which complexes with Co^2+^, slows nucleation, and directs the formation of hexagonal sheets.

VO(acac)_2_ enables V-Co partial cation exchange and diffusion imbalance, promoting the central etching required for hexagram formation.

Thus, NH_4_F + VO(acac)_2_ jointly regulate morphology.

To prepare the hexagram structure, 1.25 g NH_4_F was dissolved into 40 mL DI water. Then, 0.265 g VO (acac)_2_ and 0.436 g Co (NO_3_)_2_·6H_2_O (molar ratio 1:1.5) were dispersed in the mixed solution system. The above reagents were mechanically stirred for 20 min and heated to 200 °C with a holding time of 4 h in the autoclave. The obtained product was annealed at 500 °C for 2 h with a heating rate of 2 °C/min after washing with DI water and ethanol several times.

To investigate the agents’ effects on structure, the ratio of VO (acac)_2_ to Co (NO_3_)_2_·6H_2_O was adjusted to 0:1, 1:1, 1:1.5, 1:2, 2:1, 1.5:1, or 1:0. The amount of NH_4_F was adjusted from 0 mM to 40 mM. The pH value was adjusted from 6 to 9. The reaction time was adjusted from 2 h to 6 h. The temperature was adjusted from 160 °C to 220 °C.

After multiple experiments, the optimal experimental conditions were determined to be NH_4_F: **33.74 mM** (1.25 g in 40 mL); V:Co ratio: **1:1.5**; temperature: **200 °C**; time: **4 h**; pH: **7.0**.

### 2.2. Material Characterization

The phase was measured using a Shimadzu LabX-XRD-6000 X-ray diffraction (XRD) instrument and Cu K_α_ radiation. The morphologies of the samples were detected using a Hitachi S-4800 field-emission scanning electron microscopy (FE-SEM) attached to a Bruker QUANTAX 200 EDS and JEOL, JSM-7600F, 5 kV. The microstructure was determined by using a transmission electron microscope (TEM) (Titan G2 60–300). Elemental mapping on the selected surface morphology area was evaluated using an energy dispersive X-ray spectroscopy (EDX) instrument connected to an HRTEM. X-ray photoelectron spectroscopy (XPS) (Thermos Multi-Lab 2000 System) was used to investigate the elemental composition and chemical states.

### 2.3. Electrochemical Characterization

The electrochemical performance was investigated using coin cells and a three-electrode system. The working electrodes were fabricated by coating the active material, carbon black, and PVDF (7:2:1) on a Ni foam substrate (1 cm × 1 cm). (**mass loading:** 1.0–1.5 mg active material; **electrode area:** 1 cm × 1 cm; film **thickness:** 120–150 μm (measured after drying) and then drying overnight at 70 °C. All electrochemical measurements were performed using CHI604E and LAND CT2001A. The working electrode, reference electrode, and counter electrode were V-Co, the Ag/AgCl electrode, and the Pt plate, respectively. The electrolyte was a 6 M KOH aqueous solution. The specific capacity (C_s_, C/g) and capacitance (C_s_, F/g) associated with the voltage of the Co_3_O_4_ electrodes were calculated from the CV and GCD curves using Equations (1) and (2):

(1) $$Cs=\frac{\int I(V)dV}{mv△v}$$

(2) $$Cs=\frac{I△t}{m}$$

where *Cs* is specific capacity or capacitance (C/g or F/g), *I* is the mean response current (A), m represents the mass (g) of the active materials, *v* is scanning rate (V/s), and Δ*t* is the discharge time(s) in GCD, respectively.

The ratio of diffusion and capacitive contribution at a constant potential can be quantitatively determined using Equation (3)

(3) $$I=k_{1}v+k_{2}v^{1/2}$$

where *I* is the current (A) at voltage V, *v* is the scan rate (mV/s), and *k*_1_*v* and *k*_2_*v*^1/2^ represent the current contributions from the capacitive and diffusion-controlled reactions, respectively [12].

The charge storage mechanism was examined using the power law, as given in Equation (4)

(4) $$I=av^{b}$$

where *a* and *b* are adjustable variables, and the value of *b* can be calculated from the slope of log (*I*) – log (*v*). A value of *b* = 1 indicates that the charge storage mechanism is surface-controlled, while *b* = 0.5 indicates that it is diffusion-controlled [12].

## 3. Results and Discussion

### 3.1. Characterization

**Figure 1:**
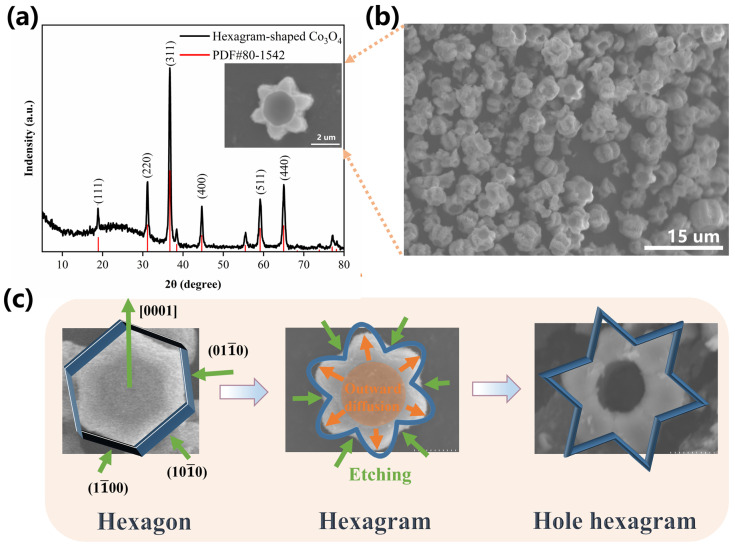
(**a**) XRD analysis of the hexagram structure, (**b**) SEM image of the hexagrams, and (**c**) growing mechanism of the hexagram structure. The inset in (**a**) is a magnified micrograph of hexagram-shaped Co_3_O_4_.

X-ray diffraction (XRD) was conducted to study the crystal patterns of the hexagram structure after annealing in the range of 5° to 80°, as displayed in Figure 1a. The XRD peaks of the hexagram structure shows (111), (220), (311), (222), (400), (422), (511), and (440) indexed peaks at 2θ of 18.9°, 31.1°, 36.8°, 38.4°, 44.6°, 55.5°, 59.2°, and 65.1°, which are consistent with Co_3_O_4_ (PDF#80-1542). The Co_3_O_4_ spinel structure exhibited a cubic crystal system space group of Fd-3m, a space group number of 227, and a lattice parameter of a = b = c = 8.08 Å [11]. Remarkably, although VO (acac)_2_ was added in solution, XRD shows that only pure hexagram-shaped Co_3_O_4_, without impurities, existed in the crystal. It can be inferred that V ions exist in amorphous form, or play the role of structure-directing agent (SDA). Figure 1b shows the micromorphology of hexagram-like Co_3_O_4_. The SEM image depicts an even distribution of hexagrammatic morphology, with each side measuring 2 μm. The inset image shows the magnified microstructure; a central circular concavity, approximately 2 μm in diameter, is present within the hexagram structure. This novel and unique hexagram-shaped morphology after tuning has rarely been reported before, and may possess robustness or desirable strain tolerance, higher specific surface, and great potential in energy storage compared with traditional bulk and hollow Co_3_O_4_-based electrode structures [6,7,8,9,10,11]. Thus, to investigate the growing mechanisms and electrochemical storage performance of hexagram-shaped Co_3_O_4_, experiments with different NH_4_F amounts, V:Co ratios, temperatures, reaction times, and pH values were conducted.

Experiments using 0 to 40 mM NH_4_F were carried out, and the results are shown in Figure S1. The morphology of the obtained product without adding NH_4_F exhibits an irregular structure (Figure S1a). The introduction of 20 mM NH_4_F leads to a morphology featuring a broken sphere, which has a hollow structure, as observed from cracked sphere shells. It could be concluded that the relatively fragile hollow sphere structure has difficultly withstanding strain forces. In addition, a small amount of bulk Co_3_O_4_ existed in the product. Using 25 mM NH_4_F reduced the number of fragile shells and increased the number of intact sphere structures, as well as the irregular bulk, as shown in the SEM images in Figure S1c. A preliminary hexagram outline shows up with 30 mM NH_4_F, along with irregular bulk, as shown in Figure S1d. Notably, more complete hexagram-shaped morphology with a depressed area in the center was seen with 35 mM NH_4_F. However, disorder returns at 40 mM, as seen in Figure S1e,f. To the best of our knowledge, micromorphology with complete structure and uniform distribution is conducive to stable ion transport and electrolyte exchange of supercapacitor electrode materials during charging and discharging, subsequently reducing internal resistance and improving electrochemical performance. Based on the above experiments, an amount of NH_4_F within the range of 30 to 35 mM is confirmed to result in a complete and uniformly distributed microstructure of hexagrammatic Co_3_O_4_. When the amount of NH_4_F is 33.74 mM (1.25 g), the microstructure of the as-prepared sample shows a novel and fascinating hexagram-like structure with no obvious incomplete growth or fragmentation (Figure 1b and inset in Figure 1a). Therefore, the optimal amount of NH_4_F for generating hexagonal hexagram morphology is identified. The analysis described above demonstrates that the NH_4_F amount greatly affects the morphology. The negative F^−^ ions from NH_4_F combine with the positive metal ions (Co^2+^) and then form metallic bonds to generate CoF_a_^(a−2)−^. This slows release of Co^2+^ ions into the solution, affecting the degradation velocity of the metal ions and reducing the rate of nucleation [13,14,15]. The structure thus becomes organized [16,17,18,19,20]. Thus, NH_4_F plays a crucial role as a structure-directing agent (SDA) in the hydrothermal reaction and induces the formation of different micromorphologies.

An experiment testing the effects of different V:Co ratios was conducted, as shown in Figure S2. Remarkably, when Co(NO_3_)_2_·6H_2_O was not included in the reaction, no precipitate was observed in the final product. This may imply that Co(NO_3_)_2_·6H_2_O plays a necessary role in the formation of the external framework structure and promotes the growth of hexagram-shaped Co_3_O_4_ crystals. In the SEM images at V:Co = 1:1, we found fragments of hexagram structures and debris with smooth edges. As the Co amount increases to 1:1.5, as shown in Figure S2b, the microstructure exhibits a regular, complete, and uniformly distributed hexagonal hexagram-shaped structure, making it an ideal and well-aligned Co_3_O_4_ electrode material for supercapacitors. When the V:Co molar ratio is 1:2, the edges of the hexagram structure become sharp and show a hollow hexagonal structure. Thus, in addition to NH_4_F as an SDA, cobalt ions play an important role in supporting the formation of hexagram structure. The morphology spectrum of the hydrothermal tests in the absence of VO(acac)_2_ is shown in Figure S2d. These conditions results in the formation of a micro-sized quasi-hexagonal shell structure with a hollow. This indicates that Co ions trends to grow into hexagonal structures and diffuse from the outer shell of the hexagram structure under NH_4_F’s directing effect, which generates the shell structure. Moreover, V ions do not participate in the synthesis reaction directly and individually, and consequently do not form corresponding crystals with Co and O, which agrees with the results of the XRD analysis. Co ions thus are the main components of the hexagram crystal structure. Nevertheless, When the V: Co molar ratio is 1.5:1, the as-synthesized samples display the coexistence of hexagonal hexagrammatic structure, spherical structure, cylindrical structure, and irregular particles, as shown in Figure S2e. Particularly, hexagram-shaped structure pieces transform into spherical structures, further evolving into a well-aligned ball-flower structure when the V:Co ratio is from 1:1.5 to 2:1. The XRD pattern and a magnified SEM image of ball-flower Co_3_O_4_ are displayed in Figure S3 and its inset. The identified crystalline structure and pure phase of the ball-flower-like structure matched the cubic phase of Co_3_O_4_ (PDF#80-1542) and are consistent with the hexagram-like structure [13]. Evidently, the micrometer-sized ball-flower structure exhibits nanosheets resulting from centripetal growth. Thus, it can be concluded that, with the facilitation effect of SDA, V ions trend to grow into spheres, and Co ions tend to grow into hexagonal sheets. The conclusion is consistent with several reports that NH_4_F directs structure into hexagonal sheets [21,22,23,24,25].

Figure S4 presents hexagram structures synthesized at a range of temperatures from 160 to 220 °C. At 160 °C, the morphology shows hexagonal bulk, and no distinct hexagram morphology was found. At 180 °C, the hexagonal bulk morphology turns into a flower-shaped morphology with a circular concavity in the flower’s center. At 200 °C, the flower-shaped structure becomes angular, resembling a hexagram, with a deeper concavity. At 220 °C, the corners of the hexagram structure are sharp and undergo a transition into a hollow structure, which shows a significant effect of hydrothermal reaction temperature on tuning the hexagonal hexagram-like structure.

Figure S5 presents the hexagram structure morphology synthesized at the optimal temperature of 200 °C at different reaction times. Hexagram-shaped bulk was identified with the 2 h reaction time and presented a minor depression located in its central area. When the reaction time increases to 4 h, the concavity becomes more pronounced, while the corners of the hexagram structure sharpen further. When the reaction time reaches 6 h, a hollow hexagram structure was found. After an 8 h reaction time, the integrity of the hollow hexagram structure was compromised. The compromised hollow hexagram structure exhibits poor structural stability (only 65% retention after 200 cycles), resulting in its collapse and subsequent inadequate cycling performance.

Figure 1b and the inset in Figure 1a show the hexagram-shaped morphology synthesized at a pH value of 7.0, and Figure S6 shows the structure at pH values ranging from 5.0 to 9.0. In acid solution (pH values ranging from 5.0 to 6.5), no hexagram structure is found by SEM. At a pH value of 6.5, fragments originating from the collapsed hexagram structure are identifiable within the SEM imagery. As the pH value increases to 7.5, deeper cycle depressions appear in the center of the hexagram structure. When the pH value reaches 8, the center of the hexagram structure exhibits a void. The hexagram structure collapses when the pH value is greater than 8.5, and only rare instances of intact hexagram structure are found when the pH value is 9. Accordingly, it can be deduced that weak alkaline solutions are conducive to hexagram structure morphology. Hydroxide ions etch the center of the hexagram structure, and an excess amount of hydroxide ions leads to a hollow structure which eventually collapses.

### 3.2. Crystal Growth Mechanism

The experimental findings highlight a significant reliance of the hexagram structure on reaction agents, showcasing the sensitivity of the morphology concerning reaction duration and temperature, which directly impact ion diffusion. Thus, we deduce that the growing mechanism of the hexagram structure is governed simultaneously by the Kirkendall effect and partial cation exchange [26,27,28,29,30]. Co and V ions tend to grow into hexagonal structures under NH_4_F’s directing effect. Under the effects of multiple agents, axial growth orientations are restricted, and the facets are etched. Herein, the hexagonal structure turned into a sharp-edged hexagram structure. V ions have a melting point of 1900 °C, which is higher than Co ions’ melting point of 1500 °C [31]. According to the Kirkendall effect, V ions diffuse faster during hydrothermal synthesis reactions, as they have a lower activation energy requirement than Co ions [32]. Hydrothermal temperatures and a gradient in the ions’ concentration provide the activation energy for the reactions and migration of ions. Meanwhile, V ions move outward in the radial direction at a lower rate. For this reason, the outward diffusion of V ions is slower than the inward diffusion of Co. Co ions thus define the boundaries that restrict V ions’ outward diffusion. V ions leave the central region before Co ions reach it, and finally the introduction of Co atoms to the outer region could balance the Co ion gradient and slow its inward diffusion [21]. With prolonged reaction time, V ions at the center of hexagram diffuse outward entirely, leading to formation of a hollow hexagram structure. This maintains the gradient balance, which results in a hollow at the center of the hexagram with sharp corners. Moreover, a higher reaction temperature facilitates diffusion, accelerating progress overall. Based on the above results, the corresponding growing mechanism is shown in Figure 1c. Similar ball-flower morphologies were found in the literature [33,34,35,36]. The mechanism can be theoretically speculated as follows: (1) the interaction of V ions and NH_4_F generates a sphere structure; (2) excess F^−^ ions activate the sphere surface to form more active sites for nucleation and growth and further promote compact adhesion between the sphere and sheets [18]; (3) V ions leave the central area, resulting in a hollow structure.

**Figure 2:**
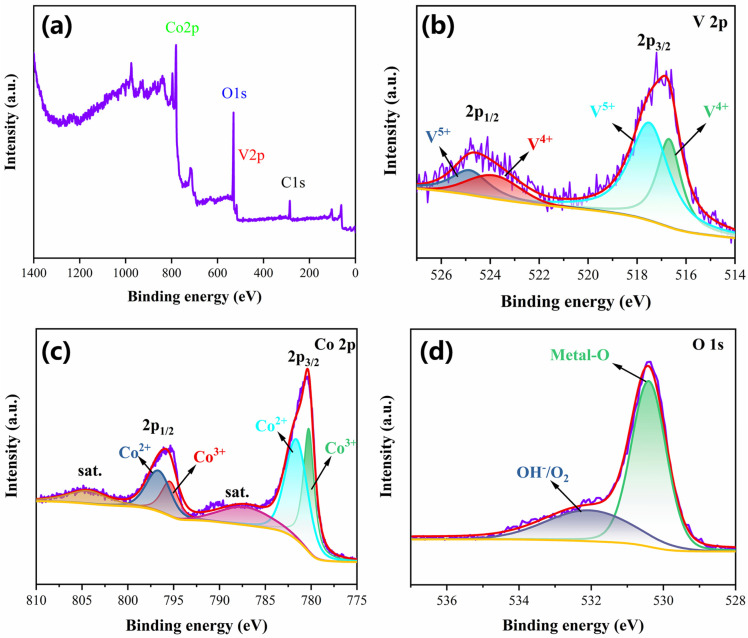
(**a**) XPS spectrum, (**b**) V 2p spectrum, (**c**) Co 2p spectrum, and (**d**) O 1s spectrum of the hexagram structure of Co_3_O_4_.

XPS measurements were conducted to analyze the oxidation states and elemental composition of hexagram-like and ballflower Co_3_O_4_ samples. Figure 2a and Figure S7a show the full XPS spectra of the hexagram structure and ball-flower structure, revealing the existence of V, Co, and O, which is consistent with XRD patterns indicating amorphous V and crystalline Co_3_O_4_. As observed in Figure 2b, two spin-orbit doublets of V 2p (2p_3/2_ and 2p_1/2_) emission spectra confirm the presence of V^4+^ (516.7 eV and 523.9 eV) and V^5+^ (517.5 eV and 523.9 eV) [37,38,39]. Two peaks correspond to Co 2p_3/2_ and Co 2p_1/2_, which can be fitted to Co^3+^ (780.3 eV and 795.3 eV) and Co^2+^ (781.6 eV and 796.0 eV), including two shakeup satellite peaks located at 787.24 eV and 804.29 eV in Co^3+^, as shown in Figure 2c [40,41,42]. In the XPS spectrum for O 1s, the fitted curve contains two oxygen distributions located at 530.41 eV and 532.05 eV corresponding to oxygen from metal-OH (M-OH) and oxygen from absorbed H_2_O molecules, as shown in Figure 2d [43,44]. The detailed XPS spectrum of the ball-flower structure is displayed in Table S1.

XPS analysis shows that V exists mainly as amorphous V^4+^/V^5+^, but the CV and CD curves do not exhibit additional redox peaks beyond typical Co^2+^/Co^3+^ and Co^3+^/Co^4+^ transitions. Electrode mass loading shows only trace V content (EDX). Thus, V primarily modulates morphology and does not significantly participate in faradic reactions.

The redox peaks arise from reversible transitions

$${\mathrm{Co}}_{3}O_{4}↔\mathrm{CoOOH}↔{\mathrm{CoO}}_{2}$$

Charge storage originates from battery-type faradic reactions (diffusion-controlled) and surface capacitive reactions.

The calculated b-values (0.64–0.66) confirm mixed kinetics.

At a representative scan rate of 1 mV s^−1^, the hexagram structure exhibits approximately 6% diffusion-controlled and 94% capacitive-controlled contribution, while the ball-flower structure shows 7% diffusion-controlled and 93% capacitive-controlled behavior.

XRD analysis indicates that there is no crystalline V-containing phase; combined with XPS and EDX, this suggests that vanadium exists in a dispersed or amorphous-like state, but not in a detectable secondary crystalline phase.

**Figure 3:**
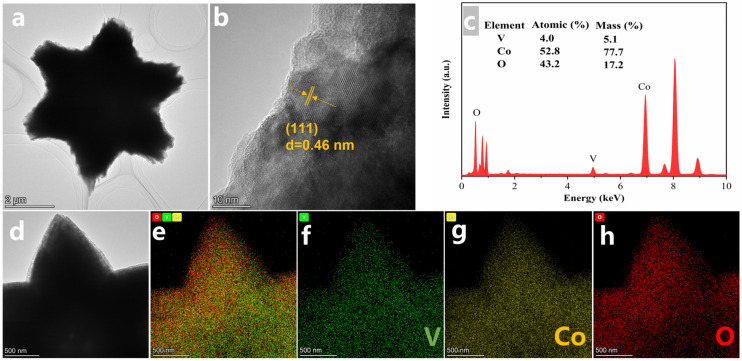
(**a**) TEM of hexagram structure, (**b**) HRTEM of hexagram structure, (**c**) EDX analysis of hexagram structure, (**d**) TEM image of a representative hexagram structure for the corresponding elemental mapping of (**e**) the full spectrum, (**f**) V, (**g**) Co, and (**h**) O for Co_3_O_4_.

TEM was used to investigate the microstructure of Co_3_O_4_ with two types of structures, as shown in Figure 3 and Figure S8. The sample exhibits complete and novel hexagram-like morphology, which is in accordance with the SEM results (inset in Figure 3a). The morphology of the hexagram tends to present a solid structure. The high-resolution TEM image displays an interplanar distance of around 0.46 nm, which is ascribed to the (111) crystal plane plotted in Figure 3b. The atomic ratio and fraction of the hexagram structure were analyzed, as shown in Figure 3c, and indicated only low V content in hexagram-like Co_3_O_4_, consistent with the previous experiments, which showed that less V and more Co resulted in better tuning of hexagram morphology. The scanning EDX results (Figure 3d–h) show that V, Co, and O are uniformly distributed throughout the hexagonal hexagram. Compared to the hexagram structure, the ball-flower structure possessed more obviously hollow and porous architecture, which may provide more ion transport paths and greater specific areas, resulting in superior capacity and performance, as depicted in Figure S8a and its inset [43]. HRTEM analysis of the ball-flower morphology, as shown in Figure S8b, indicates an interplanar distance of around 0.202 nm, which is in strong agreement with the (111) crystal plane. Also, the atomic ratio and fraction of the ball-flower structure are presented in Figure S8c, which indicates the mechanism of more V and less Co tuning ball-flower morphology under the direction of NH_4_F. Figure S8d–h reveal that V, Co, and O are also uniformly distributed throughout the ball-flower formed by interconnected nanosheets.

**Figure 4:**
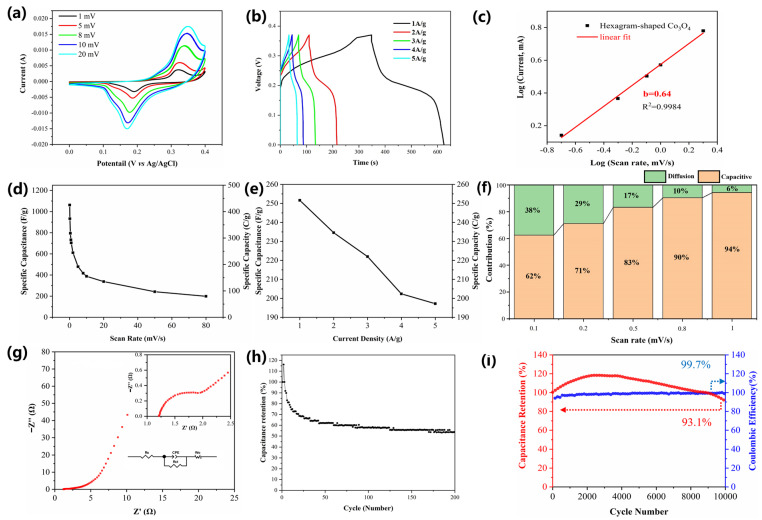
(**a**) CV curves, (**b**) GCD curves, (**c**) slope of log (IP)–log (v), (**d**) variation of specific capacity or capacitance from CV, (**e**) variation of specific capacity or capacitance from GCD, (**f**) contribution ratio of diffusion (green) and capacitive (yellow) process, (**g**) EIS curve (the insets are a magnified part of the EIS curve and the equivalent circuit), (**h**) cycling performance of the hollow hexagram-shaped structure, and (**i**) cycling performance and coulombic efficiency of the hexagram-shaped structure.

Figure 4a shows the CV curves of the hexagrammatic SC electrode in a 6 M KOH electrolyte solution, and the CV curves of the ball-flower are shown in Figure S11a. The CVs of both show vivid redox peaks inducting battery-type behavior at different scan rates, as well as good electrochemical reversibility [44,45]. The corresponding specific capacity (capacitance) and curves in Figure 4c and Figure S11c indicate that both have superior capacity. The highest specific capacities are 425 C/g (1062 F/g) and 696 C/g (1339 F/g), respectively [45,46,47]. As shown in Figure 4b and Figure S11b, GCD of the hexagram-shaped structure presents a smoother discharge platform than the ball-flower structure, at 0.15 to 0.25 V for the hexagram morphology and 0.3 to 0.35 V for the ball-flower morphology. However, the wider voltage window of the ball-flower morphology results in greater capacity than the hexagram morphology. The specific capacity curves shown in Figure 4e and the inset in Figure S11d reveal that the ball-flower structure possesses better capacity performance but more fatigued rate capability and poorer cycling performance than the hexagram structure due to its hollow and sheet-like spherical structure, which makes it easy to collapse during the continuous reaction process, unlike the steady and nearly solid hexagram morphology [48]. The highest GCD specific capacities of the hexagram and ball-flower structures are 252 C/g (680 F/g) and 563 C/g (1126 F/g), respectively.

Furthermore, the ratios of diffusion and capacitive contribution for the hexagram structure were analyzed to investigate the charge/discharge kinetics and the diffusion controlled and capacitive processes, using CV tests at various scan rates from 0.1 to 1 mV/s (Figure 4f and Figure S10). The b-value calculated for the hexagram-shaped electrode material is 0.64, and that for the ball-flower electrode material is 0.66, as shown in Figure 4c and the inset in Figure S11c. The results show that the charge storage mechanism of Co_3_O_4_ for both the hexagram structure and the ball-flower structure is a combination of diffusion control and capacitive control. The hexagram structure shows a smaller percentage of the diffusion-controlled process compared with the pure ball-flower structure. However, the electrode kinetics of the Co_3_O_4_ supercapacitor electrode material are mainly conferred by the capacitive process at a high scan rate, whose capacitive contribution reaches 94% for the hexagram morphology and 93% for the ball-flower morphology at the scan rate of 1mV/s. Based on EIS analysis (Rₛ ≈ 0.6 Ω Rct ≈ 1.8 Ω (hexagram), the diffusion slope (Warburg coefficient) indicates faster ion transport for the hexagram structure. The performances of hexagram and ball-flower structures are shown in Figure 4g and Figure S11d.

The stability of the hexagram and ball-flower structures was investigated by GCD test. As shown in Figure 4i and Figure S11c, the hexagram structure demonstrates an approximately 25% enhancement in capacity during the initial 4000 cycles, followed by a subsequent decline, which could be attributed to sustainable activation of hexagram-like Co_3_O_4_ at 1 A/g and occurs when the electrode cannot be completely soaked in the electrolyte, since it cannot be fully immersed at first [41]. It is noteworthy that its capacity retention remains at 93.1% after 10,000 cycles, because the hexagram morphology is a more stable and relatively solid structure, resulting in outstanding cycling stability during the faradic reaction and charge/discharge process, compared to the ball-flower morphology [49]. This indicates improved performance compared with several reports, such as Minakshi and his group’s TMO electrodes [50], which generally show 150 to 550 F g^−1^ and 70% to 90% retention after 5000 cycles. Our hexagram Co_3_O_4_ achieves 1062 F g^−1^ and 93.1% retention after 10,000 cycles. This will be discussed further in the Discussion. In addition, the high specific capacitance values and outstanding retention of the prepared hexagram-like Co_3_O_4_ electrode is comparable to and even higher than previously reported metal oxide-based nanostructured materials, as mentioned in Table 1. As shown in Figure S11c, the ball-flower and hexagram structures exhibit about a 5% enhancement in capacitance due to initial activation of the electrode during the first 200 cycles and incomplete soaking of electrolytes. After 1000 cycles, the ball-flower structure presents a higher than 85% capacity retention rate. However, collapse of the ball-flower structure differentiates the cycling performance due to hollow sphere structure formed by intersecting sheets and structural collapse in subsequent cycles. Cycling tests indicate the outstanding cyclic performance of the hexagram structure compared with the ball-flower structure. The coulombic efficiency (CE) of both obtained Co_3_O_4_ electrodes could be well retained after charge/discharge cycles.

## 4. Conclusions

In this work, by employing NH_4_F as a structure-directing agent (SDA), we have manipulated the morphology of Co_3_O_4_, leading to the creation of a novel hexagram-like structure with exceptional evenness of distribution. To comprehend the growth mechanism and elucidate the functions of the various agents involved, experiments were conducted under diverse conditions and with varying reagent ratios. The results indicate that, under the influence of NH_4_F as the structure-directing agent (SDA), the hexagram-shaped Co_3_O_4_ structure exhibits sensitivity to both reaction time and temperature, implying that its growth mechanism is regulated by the Kirkendall effect and involves partial cation exchange. Additionally, with the alteration of reagent ratios, Co_3_O_4_ with ball-flower morphology was synthesized successfully. Through cross-section SEM examination, the observed growth mechanisms for both the hexagram and ball-flower structures are substantiated. Lastly, electrochemical performance tests of the hexagram and ball-flower structures on SC electrodes were carried out, and the specific capacitances were 1062 and 1339 F/g, respectively. The hexagram-shaped Co_3_O_4_ structure displays exceptional SC electrode material characteristics, retaining 93.1% capacitance even after 10,000 cycles, highlighting its superior cycle performance.

## Figures and Tables

**Table 1:** Comparison of the electrochemical properties of Co_3_O_4_ nanomaterials reported in the previous literature.

Electrode Material	Specific Capacitance	Capacitance Retention	Ref.
Needle-like Co_3_O_4_/graphene	157.7 F g^−1^ at 0.1 A g^−1^	70% after 4000 cycles	[3]
Co_3_O_4_/Ni foam	1.92 F cm^−2^ at 5 mA cm^−2^	72.91% after 3000 cycles	[5]
Co_3_O_4_	658.2 F g^−1^ at 1 A g^−1^	90.4% after 5000 cycles	[6]
Gully-Network Co_3_O_4_ nanowire arrays	582.8 Cg^−1^ at 1A g^−1^	74% after 10,000 cycles	[51]
ZnO-Co_3_O_4_ core-shell heterostructure	177.0 F g^−1^ at 1.4 A g^−1^	92.8% after 10,000 cycles	[52]
ZnO/Co_3_O_4_ nano-bundle arrays graphene	198.0 F g^−1^ at 1 A g^−1^	86.5% after 5000 cycles	[53]
Porous rod-shaped CuO@Co_3_O_4_	545.5 F g^−1^ at 1 A g^−1^	88.7% after 10,000 cycles	[54]
Fe-doped Co_3_O_4_ Nanosheet floral clusters	1 A g^−1^ at 680 F g^−1^	84.67% after 5000 cycles	[55]
Hollow and octahedral Co_3_O_4_/Fe_2_O_3_	659.7 F g^−1^ at 0.5 A g^−1^	63.7% After 6000 cycles	[56]
Mesoporous Co_3_O_4_ nanorods	261 F/g at 0.25 A/g	---	[57]
CuO-NiO-Co_3_O_4_ nanocomposites	262 Fg^−1^ at 1 Ag^−1^	84.9% after 5000 cycles	[58]
**Hexagram Co_3_O_4_**	1062 F g^−1^	93.1% retention at 10,000 cycles	
**Ballflower Co_3_O_4_**	1339 F g^−1^	85% retention at 1000 cycles	

## Data Availability

Data is contained within the article or Supplementary Materials.

## Supplementary Materials

The following supporting information can be downloaded at: https://www.mdpi.com/article/10.3390/nano16030162/s1.
