# Supplementary material for: NH4F and VO (Acac)2 Tuning of Hexagram-Shaped Co3O4 Morphology for High-Performance Supercapacitor Electrodes
Source: Nanomaterials (Basel). 2026 Jan 26;16(3):162. doi: 10.3390/nano16030162 (PMC12900016; doi:10.3390/nano16030162)
Supplement: Supplementary file 1 [file nanomaterials-16-00162-s001.zip › nanomaterials-3892696-supplementary.pdf]

# Supplementary Material

# $NH_4F$ and $VO(acac)_2$ tuning hexagram-shaped $Co_3O_4$ morphology for high performance supercapacitor electrodes

*Huanping Yang[a,*], Zhiguo Zhang[a,b], Ziming Fang[a], Yutian Zhao[c], Bitao Xiong[a], Xiaoli Lang[a], Yanting Shen[a], Xing'ao Li[a,d], Yan Wang[a]*

[a] School of Science, Zhejiang University of Science and Technology, No.318, Liuhe Road, Xihu District, Hangzhou 310023, PR China
[b] Tsing Bosch(Zhuhai) Technology Co., Ltd
[c] School of Textile Science and Engineering, Xi'an Polytechnic University, No.58, Shan Gu Avenue, Lintong District, Xi'an 710600, PR China
[d] School of Science & New Energy Technology Engineering Laboratory of Jiangsu Province, Nanjing University of Posts and Telecommunications (NJUPT), Nanjing, 210023, China

[*] Corresponding author: Huanping Yang
***E-mail** address:*
Huanping Yang: hpyang@zust.edu.cn

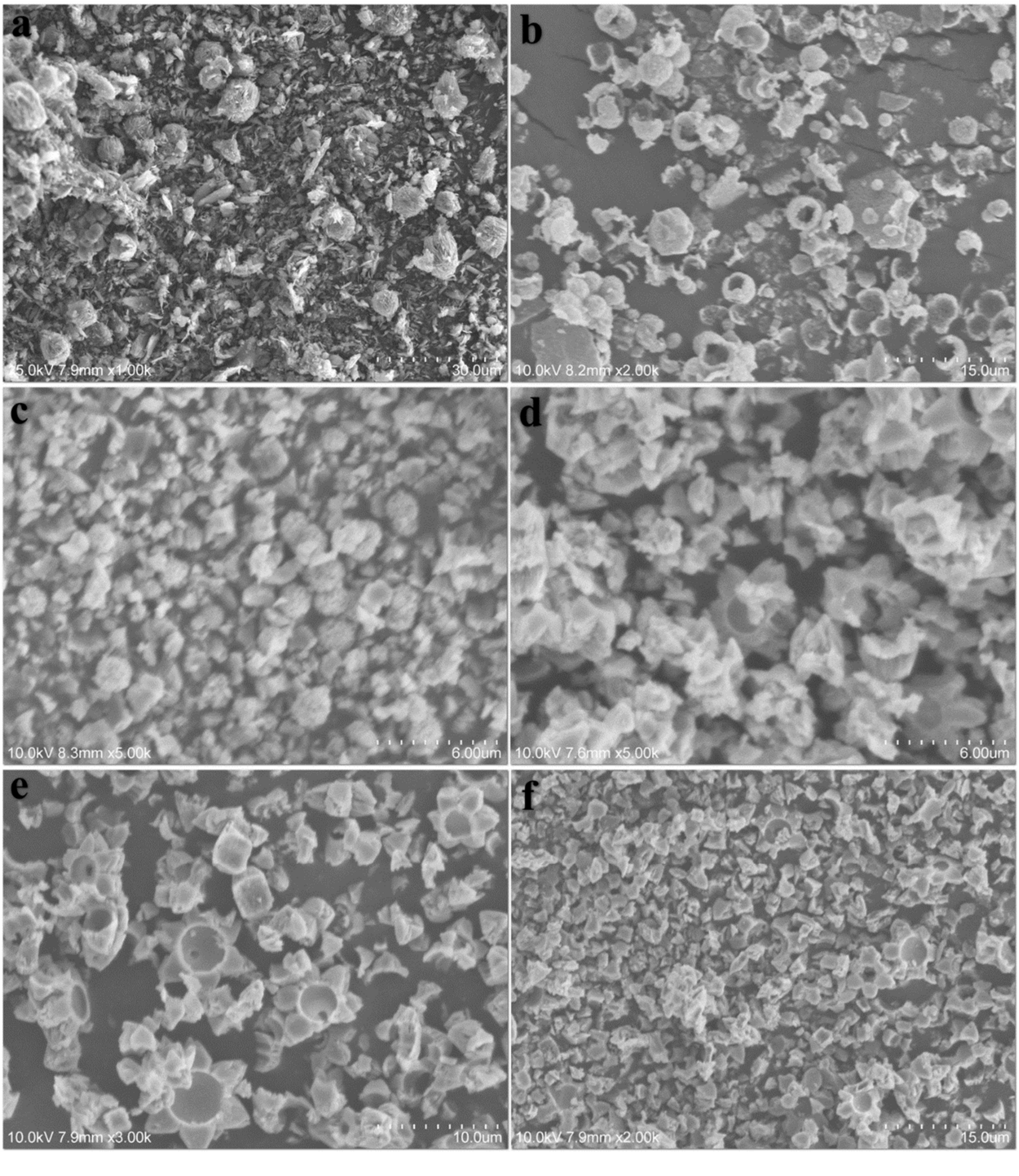


**Figure S1.** As-obtained samples of the amount of $NH_4F$ tuning at (a) 0 mM, (b) 20 mM, (c) 25 mM, (d) 30 mM, (e) 35 mM, (f) 40 mM

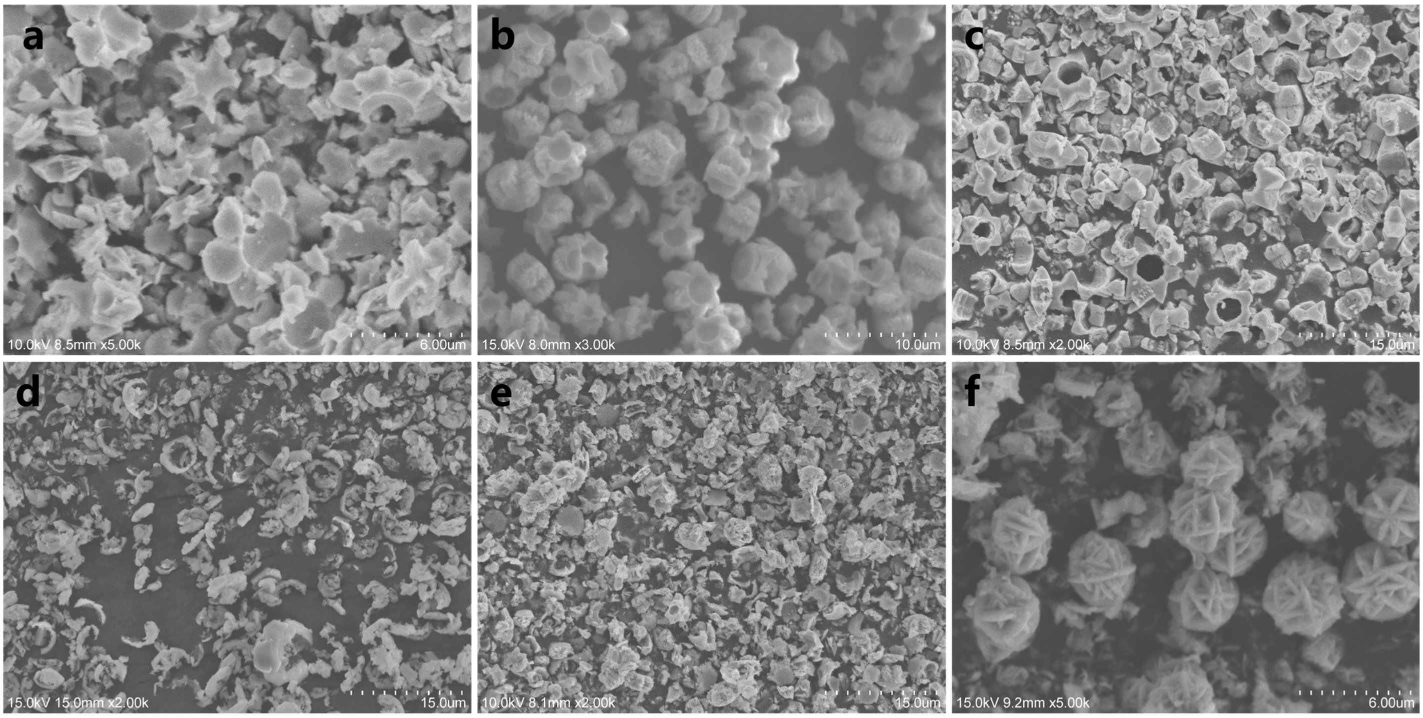


**Figure S2.** As-obtained samples of V:Co ratio tunning at (a) 1:1, (b) 1:1.5, (c) 1:2, (d) 0:1, (e) 1.5:1and (f) 2:1 for ball-flower $Co_3O_4$.

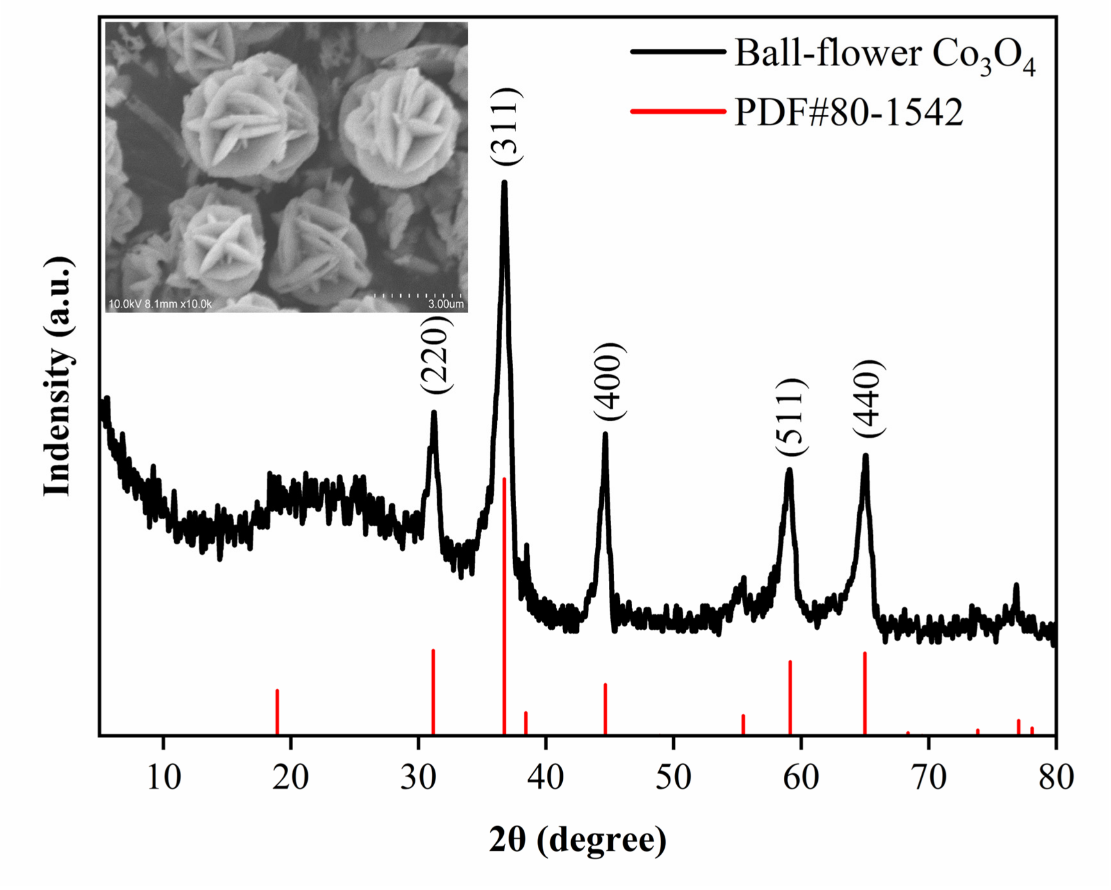


**Figure S3.** XRD of ball-flower structure

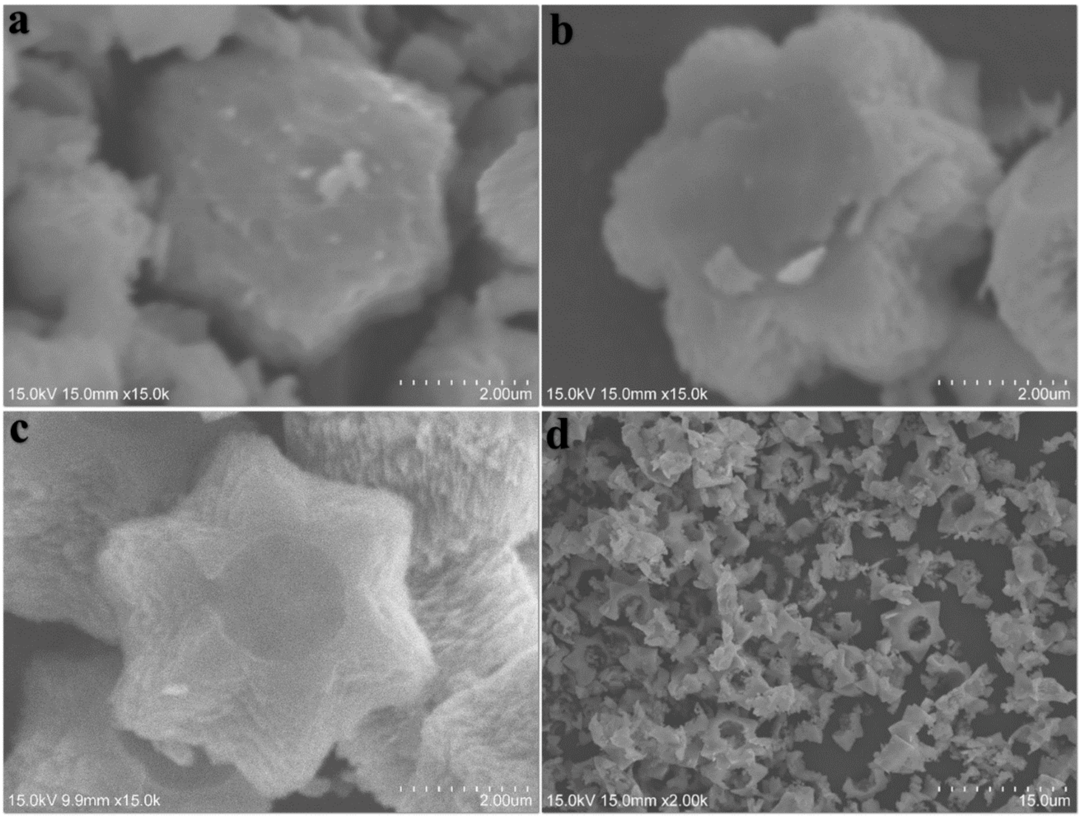


**Figure S4.** (a) As-obtained samples at the reaction temperature of 160℃, (b) 180℃, (c)200℃, (d) 220℃

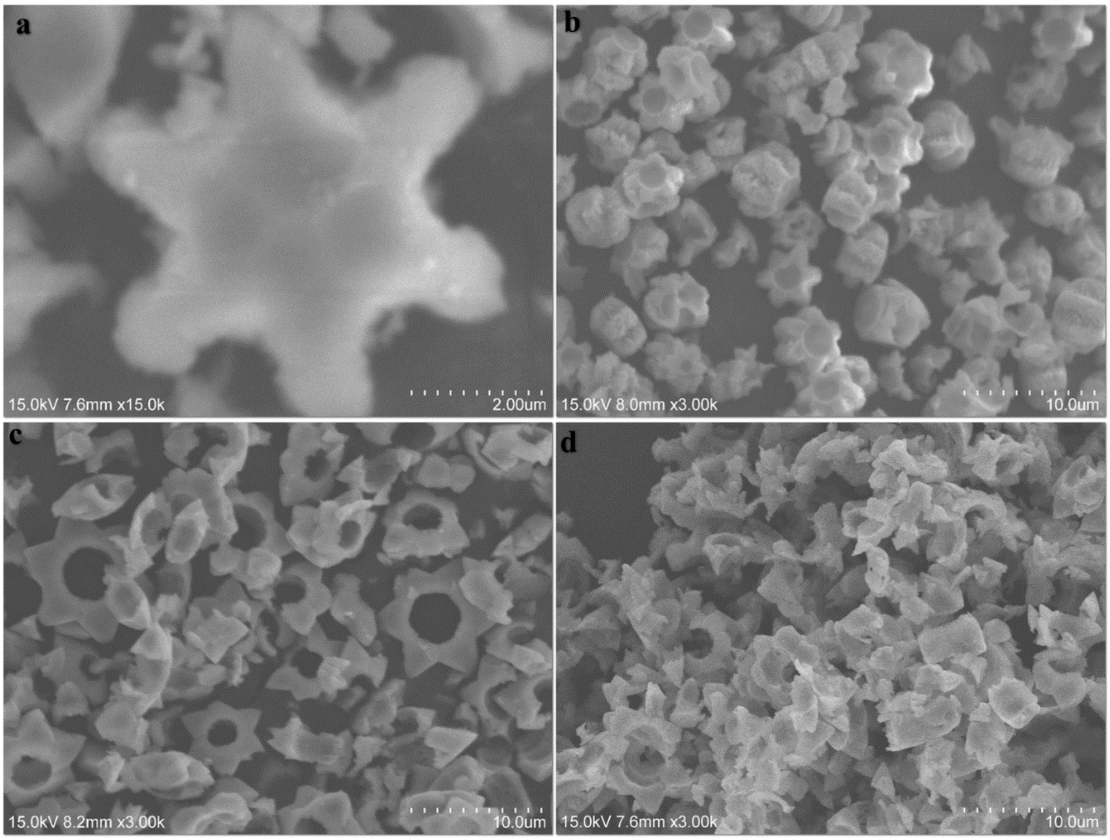


**Figure S5.** As-obtained samples at the reaction time of (a) 2 h, (b) 4 h, (c) 6 h, (d) 8 h

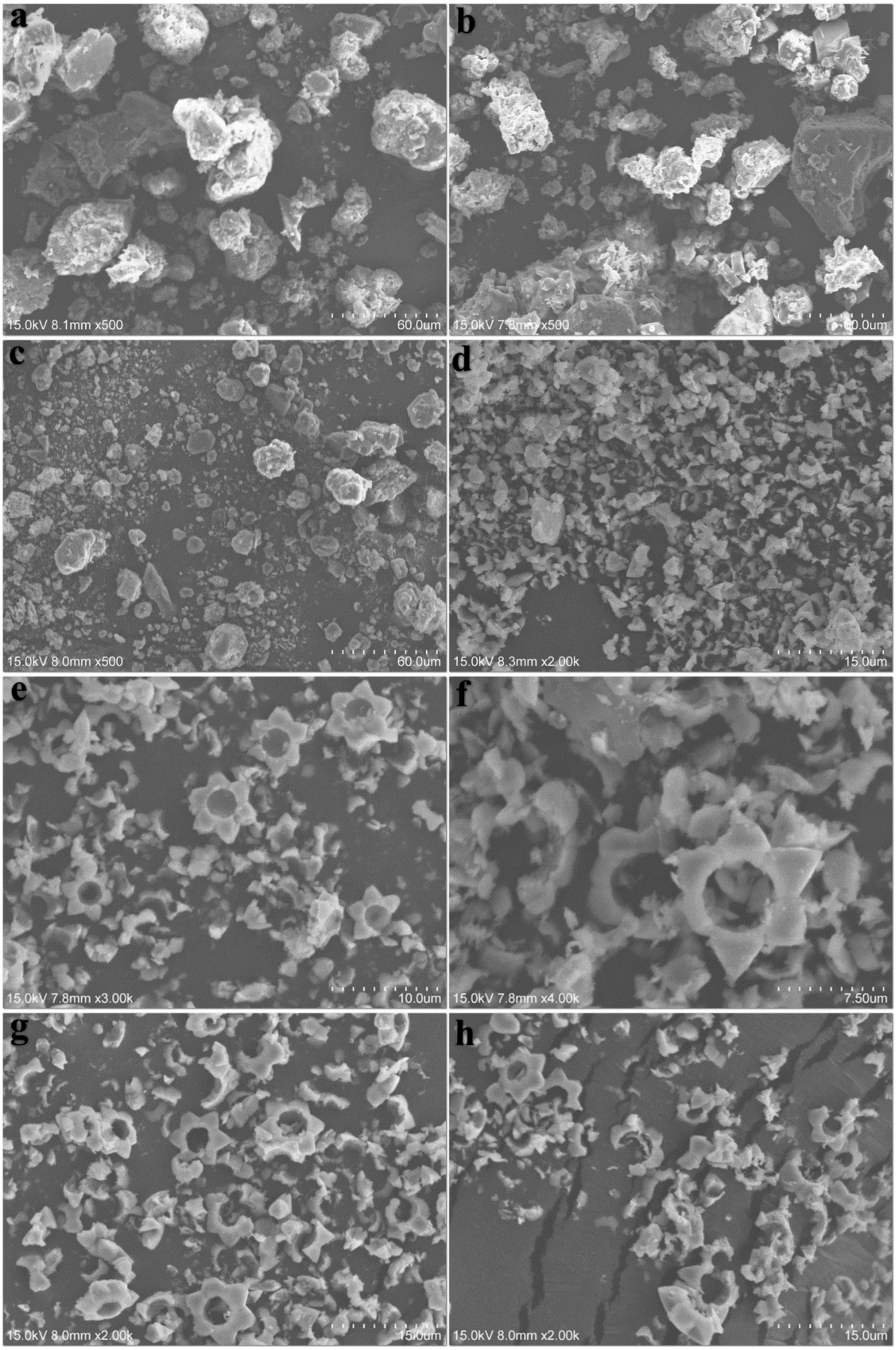


**Figure S6.** pH value of (a) 5.0 (b) 5.5 (c) 6.0 (d) 6.5 (e) 7.5 (f) 8.0 (g) 8.5 (h) 9.0

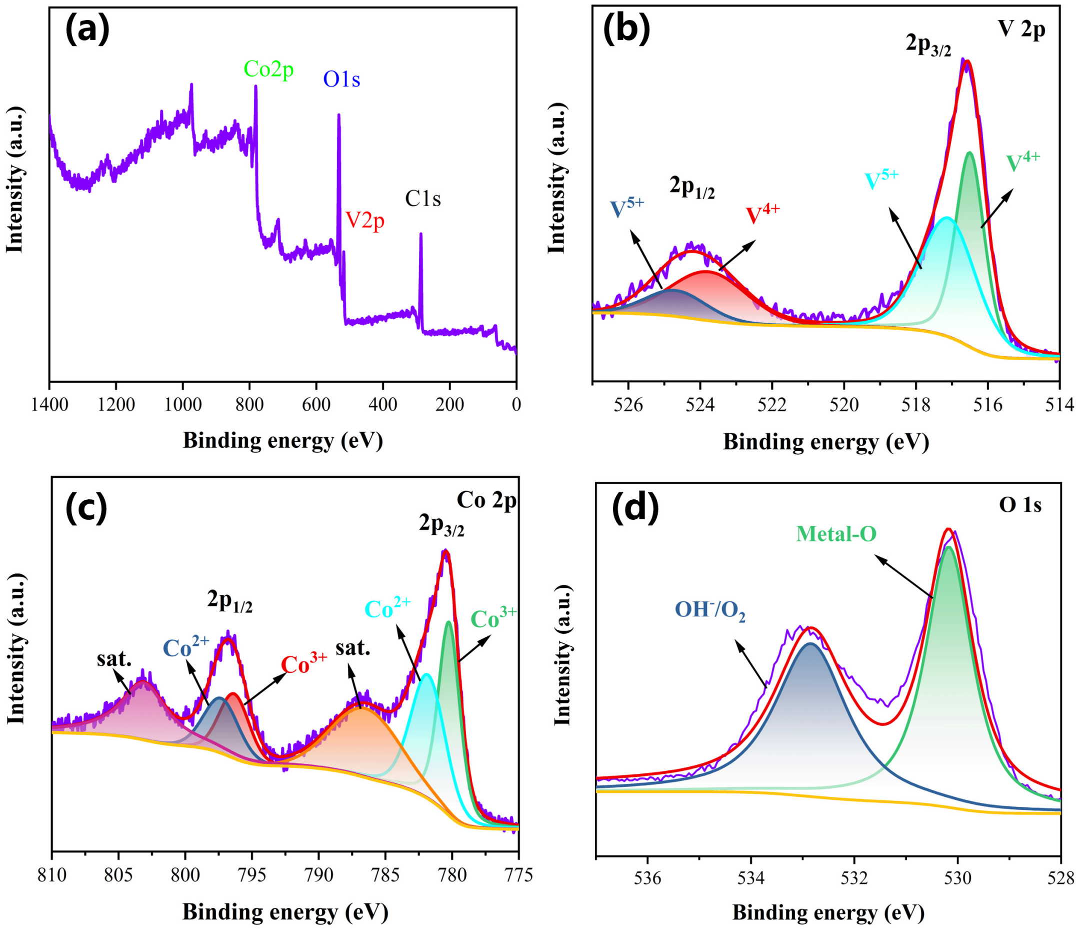


**Figure S7.** (a) XPS spectrum, (b) V 2p spectrum, and (c) Co 2p spectrum, and (d) O 1s spectrum of ball-flower structure for $Co_3O_4$.

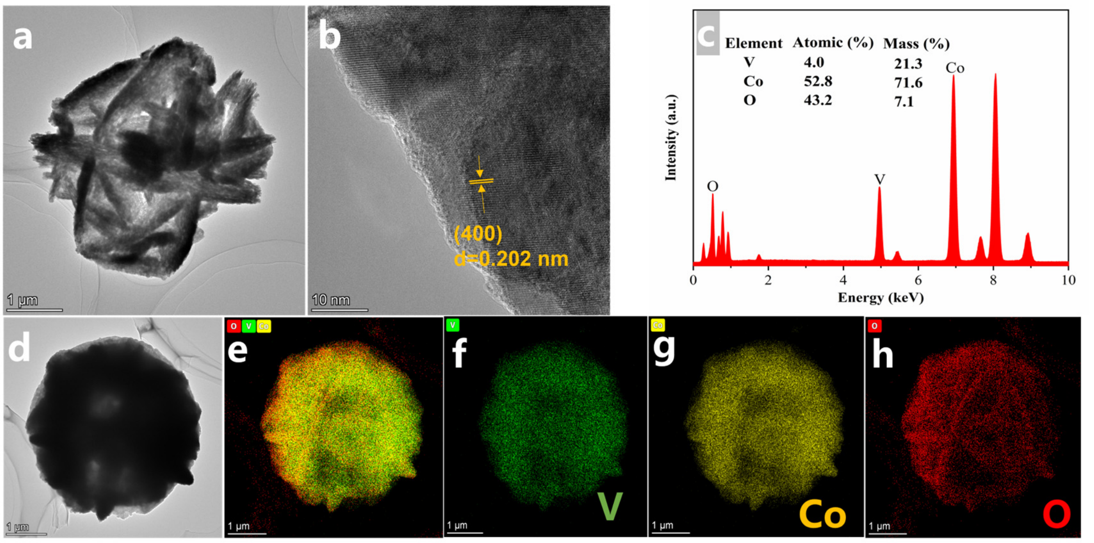


**Figure S8.** (a) TEM of ball-flower structure, (b) HRTEM of ball-flower structure (c) EDX analysis of ball-flower structure, (d) TEM image of a representative ball-flower structure for the corresponding elemental mapping of (e) full spectrum, (f) V, (g) Co, and (h) O for $Co_3O_4$

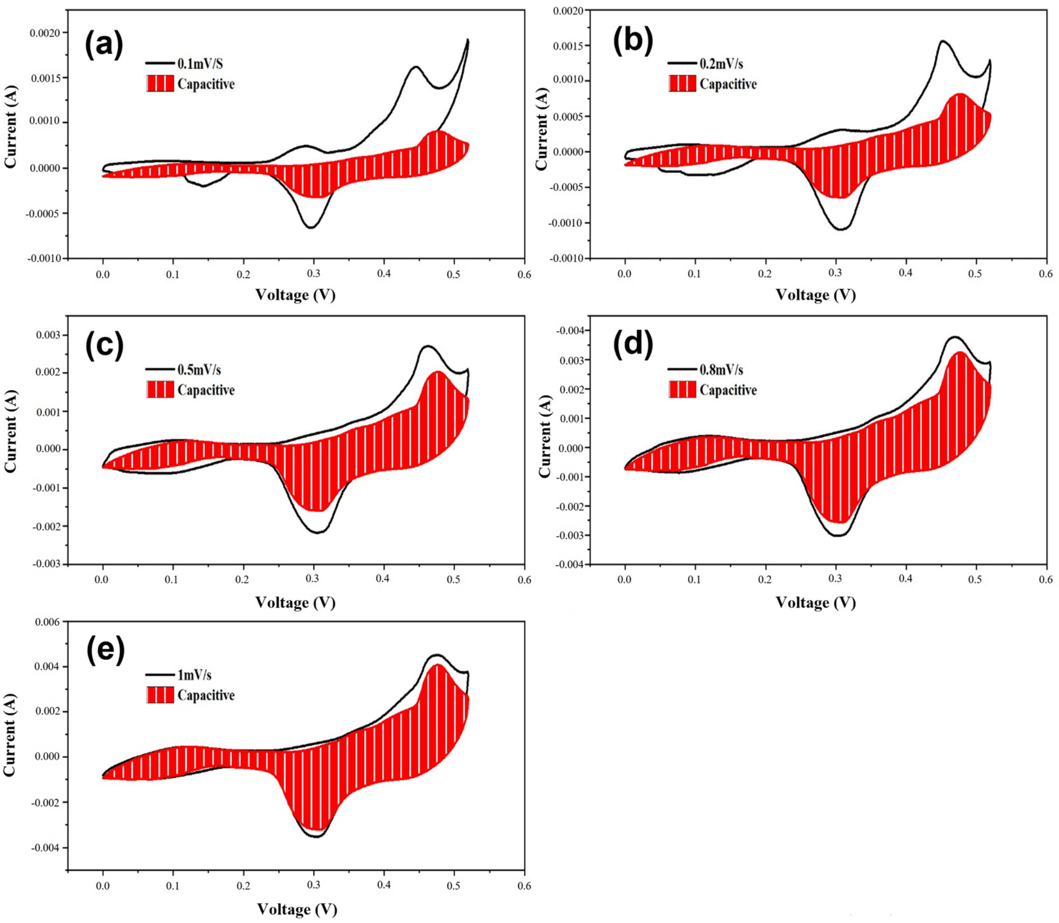


**Figure S9.** Separation of the surface and diffusion controlled currents at (a) 0.1 mV/s, (b) 0.2 mV/s, (c) 0.5 mV/s, (d) 0.8 mV/s, (e) 1 mV/s of ball-flower structure.

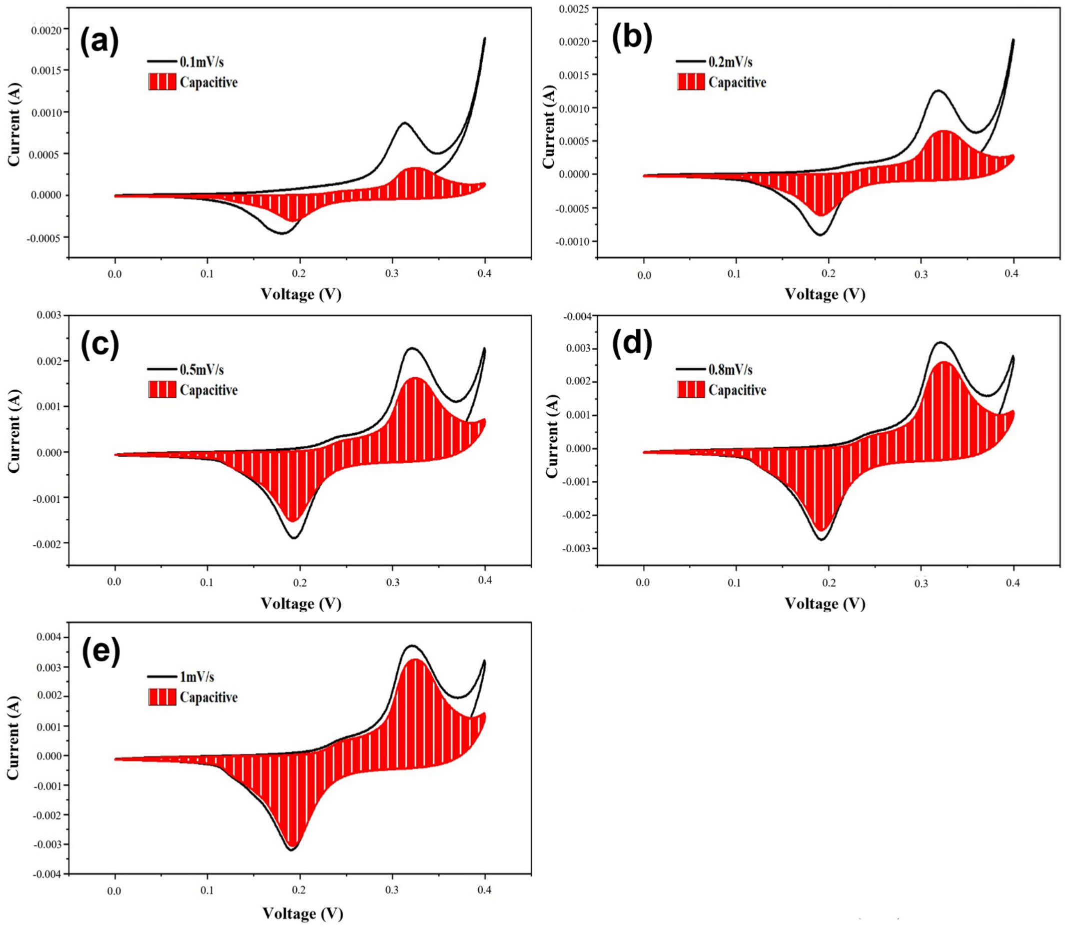


**Figure S10.** Separation of the surface and diffusion controlled currents at (a) 0.1 mV/s, (b) 0.2 mV/s, (c) 0.5 mV/s, (d) 0.8 mV/s, (e) 1 mV/s of hexagram-shaped structure.

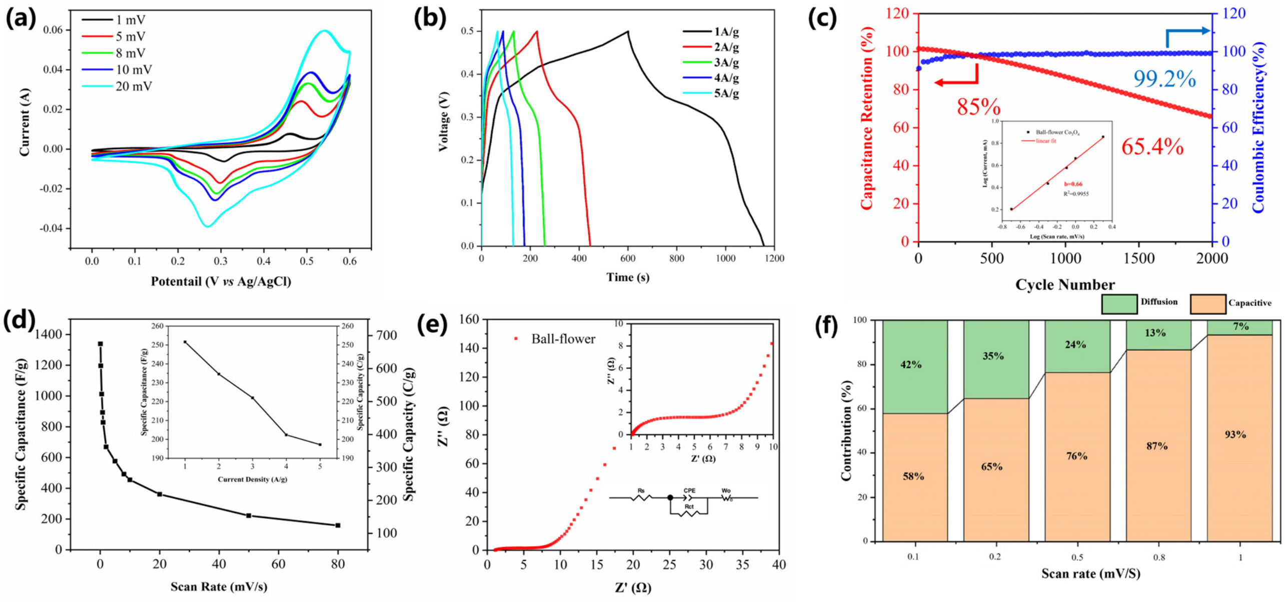


**Figure S11.** (a) CV curves, (b) GCD curves, (c) Cycling performance and coulombic efficiency (the insert is the slope of log (IP)-log(v)), (d) The variation of specific capacity or capacitance from CV (the insert is the variation of specific capacity or capacitance from GCD), (e) EIS curve (the inserts are respectively magnified part of EIS and equivalent circuit), (f) Contribution ratio of diffusion (Green) and capacitive (Yellow) process of ball-flower structure.

**Table S1.** The detailed XPS spectrum of ball-flower structure.

| Spin-orbit | Binding energy | Value |
|---|---|---|
| Co $2p_{3/2}$ | 780.25eV | $Co^{3+}$ |
| | 781.9 eV | $Co^{2+}$ |
| Co $2p_{1/2}$ | 796.45 eV | $Co^{3+}$ |
| | 797.45 eV | $Co^{2+}$ |
| Sat. | 786.85 eV | $Co^{3+}$ |
| Sat. | 804.29 eV | $Co^{3+}$ |
| V $2P_{3/2}$ | 516.49 | $V^{4+}$ |
| | 517.09 | $V^{5+}$ |
| V $2P_{1/2}$ | 523.75 | $V^{4+}$ |
